# MySeq: privacy-protecting browser-based personal Genome analysis for genomics education and exploration

**DOI:** 10.1186/s12920-019-0615-3

**Published:** 2019-11-27

**Authors:** Michael D. Linderman, Leo McElroy, Laura Chang

**Affiliations:** Department of Computer Science, Middlebury College, Middlebury, VT USA

**Keywords:** Personal Genome analysis, Genomics education, Web application

## Abstract

**Background:**

The complexity of genome informatics is a recurring challenge for genome exploration and analysis by students and other non-experts. This complexity creates a barrier to wider implementation of experiential genomics education, even in settings with substantial computational resources and expertise. Reducing the need for specialized software tools will increase access to hands-on genomics pedagogy.

**Results:**

MySeq is a React.js single-page web application for privacy-protecting interactive personal genome analysis. All analyses are performed entirely in the user’s web browser eliminating the need to install and use specialized software tools or to upload sensitive data to an external web service. MySeq leverages Tabix-indexing to efficiently query whole genome-scale variant call format (VCF) files stored locally or available remotely via HTTP(s) without loading the entire file. MySeq currently implements variant querying and annotation, physical trait prediction, pharmacogenomic, polygenic disease risk and ancestry analyses to provide representative pedagogical examples; and can be readily extended with new analysis or visualization components.

**Conclusions:**

MySeq supports multiple pedagogical approaches including independent exploration and interactive online tutorials. MySeq has been successfully employed in an undergraduate human genome analysis course where it reduced the barriers-to-entry for hands-on human genome analysis.

## Background

The growing deployment of genome sequencing in research, clinical and commercial contexts is creating a corresponding need for more effective and scalable genomics pedagogy for both providers and patients/participants [1–10]. New genomics curricula are in development to provide students hands-on experience tackling the increased scale and complexity of genome sequencing data [11–19]. However the complexity of genome informatics is a recurring challenge, even in settings with substantial computational resources and expertise [20, 21], creating a barrier to wider implementation of experiential genomics education [22]. Reducing the need for command-line and other specialized software will increase student access to hands-on genome analysis experiences.

Web applications can provide an easier-to-use alternative to command-line and other specialized software. In a traditional “server-side” web application the genomic analyses would be performed on a remote server. Modern web technologies, however, enable genomic analyses to be performed entirely in the user’s web browser. This “client-side” approach can provide the same ease-of-use while protecting the privacy of users’ sensitive genomic data (no data is uploaded to a remote server) and minimizing the infrastructure required for hands-on genomic analysis (no need for an application server). Ensuring users maintain control over their genomic data is a particularly important feature for the growing number of courses in which students analyze their own genomic data [11, 23–27].

GENOtation (formerly named the Interpretome) [28] is a web browser-based genome interpretation tool developed to support students’ analysis of their microarray genotyping data [26]. GENOtation loads the genotyping data locally from the user’s computer and performs the analyses exclusively within the browser. GENOtation is not designed, however, for use with variant call format (VCF) files commonly produced by whole exome and genome sequencing (WES/WGS). DNA Compass [29] employs a similar browser-based model for querying locally-stored VCF files downloaded from the DNA.Land digital biobank [30] (or other sources) and linking those variants to public databases, but does not implement other analyses. The iobio suite [31, 32] includes applications for combined browser and server-based analysis of locally-stored or remotely-available VCF files but is focused on filtering for putative disease variants. Web-based genome browsers and pileup viewers, such as the UCSC Genome Browser [33], JBrowse [34], igv.js [35] and pileup.js [36], can display remotely-available coordinate-indexed VCF files without additional software and some tools can also display locally-stored VCF files (e.g., igv.js and JBrowse), but a genome browser only provides limited variant analysis functionality (primarily query by genomic region).

Here we present MySeq, a freely available open-source web application, inspired by GENOtation, DNA Compass and the iobio suite, which is designed to meet the unique needs of experiential genomics pedagogy, including students analyzing their own genomic data. Motivated by our own medical genomics teaching experiences [27], MySeq enables students to get started performing hands-on genome analyses with just “one click”. MySeq can query WGS-scale Tabix-indexed VCF files, either stored locally on the user’s computer or remotely available via HTTP(S), without needing to load the entire file. Similar to GENOtation and DNA Compass, all analyses are performed within the browser without sending any genotypes to a remote server to protect the privacy of users’ genomic data. MySeq implements a variety of analyses including variant querying and annotation, physical trait prediction, pharmacogenomics (PGx), polygenic disease risk and ancestry visualization to provide representative pedagogical examples. We describe the implementation of MySeq and our experience employing MySeq in an intensive undergraduate human genome analysis course.

### Implementation

**Fig. 1:**
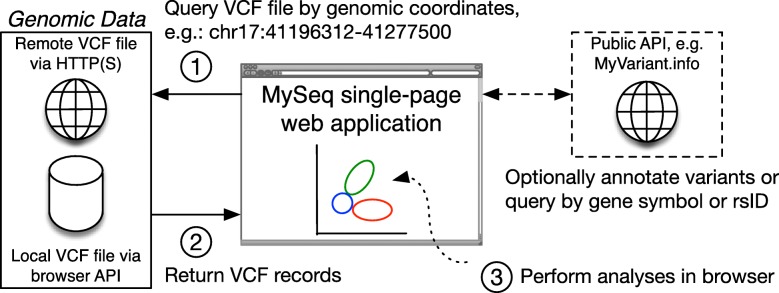
Overview of dataflow in MySeq. The MySeq single-page web application performs personal genome analyses in the user’s web browser. (1) MySeq components query a locally stored or remotely available VCF file by genomic coordinates. (2) Internally MySeq uses the Tabix index to fetch and parse only the portion of the file containing variants in the query region. (3) MySeq further analyzes the VCF records entirely in the browser (e.g. displays the genotypes to the user, performs ancestry analysis, etc.). Optionally MySeq can utilize the publicly available MyVariant.info and MyGene.info APIs [37] to annotate variants or translate gene symbols or rsIDs to genomic coordinates for queries (e.g. query for all variants in *BRCA1*), but does not send any genotypes to a remote server

MySeq is a single-page web application implemented in JavaScript ES6 with React.js. Figure 1 shows an overview of the dataflow within MySeq. All analyses begin with a compressed and Tabix-indexed VCF file [38]. The user selects a local VCF and its accompanying index file, enters a HTTP(S) URL for a VCF file or selects a preconfigured public genome (NA12878 Genome in a Bottle callset [39]). Alternately the URL of the VCF file can be provided as a URL query parameter. MySeq loads the entire Tabix index (typically 1 MB or less in size) into the browser’s memory and uses that index to efficiently determine and load just the small portion of the VCF file containing the variants needed for an analysis. The index calculations, fetch, decompression and VCF parsing are performed entirely within the browser.

MySeq supports the GRCh37/hg19 and hg38 reference genomes and VCF files with multiple samples. The analyses, and particularly the variant annotation functionality, assumes the VCF file is normalized to make all variants bi-allelic, left-aligned and trimmed [40]. A normalization script is included in the source repository to assist in preparing data for use with MySeq.

**Fig. 2:**
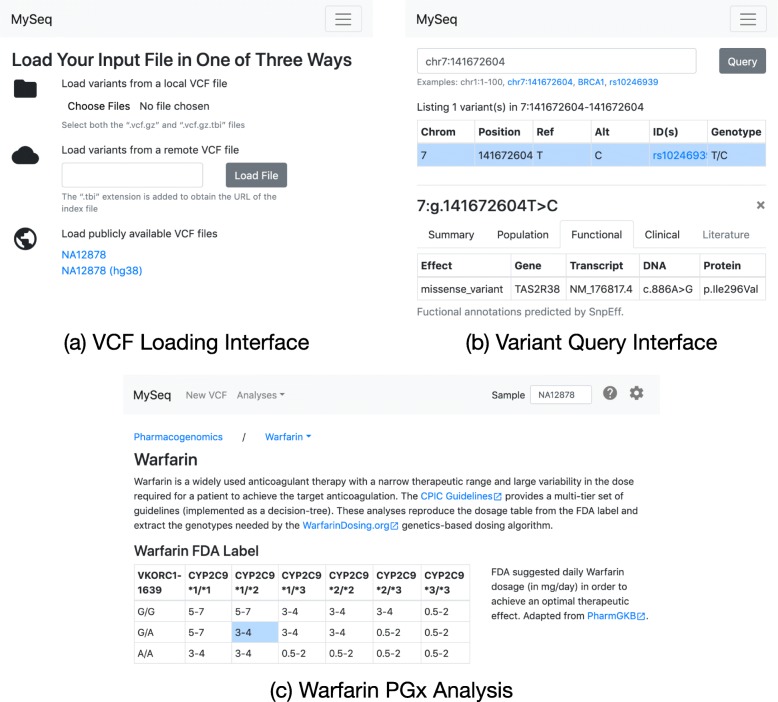
Example of MySeq VCF loading, variant query and PGx interfaces. **a** The user can load data is several ways, including pre-configured publicly available genomes. **b** Having loaded NA12878’s genome, the user’s query of chr7:141672604 returned one overlapping variant 7:g.141672604 T > C for which NA12878 is heterozygous. The user clicked on the variant to obtain functional and other annotations from MyVariant.info [37]. (c) Via the “Analyses” dropdown in the header bar (shown fully expanded in the larger screenshot), the user can launch other analyses, e.g. extract variants associated with Warfarin dosing

Table 1 describes the functionality currently available in MySeq. Each analysis is implemented as a separate React component. Figure 2 shows the user interface for the VCF loading, variant query and Warfarin PGx components as examples. An analysis component typically queries for one or more variants by genomic position when it loads, dynamically updating the user interface (UI) as the data is returned. The queries are performed in a separate web worker to not block the UI. Since many analyses use similar methods, e.g. mapping the genotypes for a variant to the corresponding phenotypes, a set of shared analysis components are provided for common operations. New analyses can be readily composed from these building blocks.

**Table 1:** Description of current MySeq functionality

Category	Current Capabilities
Query	Query for variants by genomic position, rsID and gene name. Obtain comprehensive annotations, e.g. amino acid translation, allele frequency, etc., for each variant from MyVariant.info [37].
Physical Traits	Predict phenotypes for physical traits, e.g. taste PTC as bitter, based on the genotypes of one or more associated variants.
Pharmacogenomics (PGx)	Report genotypes and associated phenotypes based on CPIC guidelines and/or the drug label for Simvastatin and Warfarin.
Polygenic Disease Risk	Predict polygenic disease risk for Type 2 Diabetes using a likelihood ratio-based approach [41], and report the *APOE* ε4 genotype and associated risk data for Alzheimer Disease.
Ancestry	Scatter plot visualization of the top two principal components using loadings for 496 ancestry informative markers (AIM) [42] computed with the POPRES resource [43].

MySeq does not require its own application-specific server; any HTTP(S) server that supports serving file ranges can be used with MySeq (e.g. Apache or a service like Amazon AWS). MySeq uses the publicly available MyVariant.info API [37] to annotate variants with the predicted amino acid translation, population frequency, links to public databases like ClinVar and other data, and the MyVariant.info and MyGene.info APIs to translate dbSNP rsIDs and gene symbols to genomic coordinates for queries. Only site-level data, e.g. variant position and alleles, and not genotypes (i.e. the alleles present in a specific sample) are sent to a remote server to maintain the privacy of the user’s genomic data. The user can optionally block the use of third-party APIs.

The user selects among the available analyses using “client-side routing” so that each analysis component has a unique URL (switching between analyses within the application does not require reloading the VCF file index). By providing a URL to a remote VCF file as a query parameter to an analysis URL, instructors (and others) can distribute links to a specific analysis of specific data.

## Results

The complexity of genome informatics, and particularly the extensive use of command-line software tools, creates barriers to the wider adoption of experiential genomics education. Creating sustainable genomics pedagogy that can be used in many different educational settings, including those with fewer resources, will require minimizing the need for specialized software and other computational infrastructure [44]. Motivated by the needs we observed in our own genomics teaching we developed MySeq to: 1) enable hands-on personal genome analysis using only the learner’s web browser; 2) ensure users can maintain complete control over their genomic data by storing it locally on their computer; and 3) support diverse pedagogy, including independent exploration, structured laboratory exercises and interactive demos.

We employed MySeq in an intensive undergraduate human genome analysis course. Students analyzed both anonymous reference data (the Illumina Platinum Genomes NA12878 trio [45]) and identified personal genome sequencing data individuals had made publicly available via OpenHumans.org [46]. The VCF files were made available via HTTPS on an institutional file server enabling students to get started just by clicking on a link to MySeq that automatically loaded the relevant genome. No file downloads, software installation or other preparatory steps were required.

Students made extensive use of the query functionality to perform their own analyses as part of an independent final project. Example uses included finding and annotating possible disease-causing variants (e.g. in known disease genes) and retrieving the genotype for variants previously reported in the literature. Students completed instructor-created laboratory exercises, e.g. predicting ABO blood group or comparing polygenic disease risk for parents and children, using the relevant scientific literature and links to specific variant queries or other MySeq analyses. These links, or even the MySeq application itself, can be embedded into another webpage to create online demos. An example “demo” that embeds MySeq (via an iframe) and IGV.js [35] to predict whether NA12878 tastes the chemical PTC as bitter (a popular in-class experiment) is available at https://go.middlebury.edu/myseq-demo. Several similar demos using MySeq were integrated into the course materials as interactive complements to the lecture slides and other course materials.

MySeq reduced the computational barriers to learning in this course. The instructor could distribute links to pre-configured analyses of specific data for laboratory exercises and demos that students could use immediately without needing to install or learn to use additional software packages. Instead of just being static demonstrations, these interactive exercises were the starting point for students’ independent analyses (again with no additional software required).

The browser-based approach introduces limitations: the scale of the analyses are restricted to an amount of data that can be reasonably downloaded and an amount of computation that be performed within the browser, and most existing genome analysis software would be need to be ported (and likely extensively modified) to work in the browser environment. However, as MySeq and other browser-based tools show, sophisticated analyses are possible, even within those limitations. The flexibility and ease-of-use of “client side” web applications make this an attractive approach for expanding access to experiential genomics education.

By supporting both locally stored and remotely available VCF files from within a browser-based tool, MySeq can take advantage of the ease-of-use of a web application while ensuring users can maintain control of their data by only storing it locally. Simply storing data locally, however, does not guarantee security and privacy. MySeq does not provide additional encryption beyond that employed by the user and thus is not a substitute for implementing data security best practices, such as local data encryption.

## Conclusion

The growing deployment of genome sequencing in research, clinical and commercial contexts is creating a corresponding need for a more genomically literate workforce and populace. To meet that need we must improve genomics education at all levels. We define “student” broadly. Patient/participant genomic literacy is equally important to the effective application of genomic testing [47]. With many patients/participants now able to obtain their own genomic testing data for further self-directed analysis [48–51], we see a critical need to offer hands-on genomic education to the general public. The most useful pedagogical approaches will be those that can be readily adapted to other educational settings, including those outside traditional academic medical centers, with fewer specialist, infrastructure, and financial resources.

MySeq is not intended however to diagnose, prevent or treat any disease or condition (including to predict a person’s response to specific medications). That warning is displayed within the application when loading a VCF file and in the documentation. At present the regulatory “picture” for “third party” tools is unclear and evolving (see [52] for a recent review). Similar to GENOtation [53], the purpose of MySeq is not to perform third-party interpretation, instead MySeq is intended as a hands-on pedagogical tool for learning about how genome analyses are performed.

Here we described MySeq, a single page web-application for personal genome analysis designed to support experiential genomics education. By replacing command-line and other specialized personal genome analysis software with an easy-to-deploy and easy-to-use web application, MySeq makes hands-on personal genome analysis more accessible for students of all kinds. We hope that such a tool will contribute to the larger effort improve the availability and efficacy of genomics education for providers and patient/participants alike.

## Availability and requirements

Project name: MySeq.

Project home page: https://github.com/mlinderm/myseq, https://go.middlebury.edu/myseq

Operating system(s): Platform independent.

Programming language: JavaScript.

Other requirements: None.

License: Apache 2.

## Data Availability

The datasets analyzed during the current study are available within the application, https://go.middlebury.edu/myseq, from Genome in a Bottle, ftp://ftp-trace.ncbi.nlm.nih.gov/giab/ftp/release/NA12878_HG001/, the European Nucleotide Archive, https://www.ebi.ac.uk/ena/data/view/PRJEB3381, or at OpenHumans, https://www.openhumans.org.

## Abbreviations

PGT: Personal Genomic Testing
PGx: Pharmacogenomics
VCF: Variant Call Format
WES: Whole Exome Sequencing
WGS: Whole Genome Sequencing
